# Real-world landscape transition of death causes in the immunotherapy era for metastatic non-small cell lung cancer

**DOI:** 10.3389/fimmu.2022.1058819

**Published:** 2022-11-11

**Authors:** Yijun Wu, Zhuoran Yao, Jianhui Zhang, Chang Han, Kai Kang, Ailin Zhao

**Affiliations:** ^1^ Department of Thoracic Oncology, Cancer Center, and Laboratory of Clinical Cell Therapy, West China Hospital, Sichuan University, Chengdu, China; ^2^ Institute of Biomechanics and Medical Engineering, School of Aerospace, Tsinghua University, Beijing, China; ^3^ Department of Cancer Center, West China Hospital, Sichuan University, Chengdu, China; ^4^ Department of Hematology, West China Hospital, Sichuan University, Chengdu, China

**Keywords:** death cause analysis, immunotherapy, non-small cell lung cancer, metastatic disease, real world data

## Abstract

**Background:**

With approval of anti-PD-1/PD-L1, metastatic non-small cell lung cancer (NSCLC) has entered the era of immunotherapy. Since immune-related adverse events (irAEs) occur commonly in patients receiving anti-PD-1/PD-L1, the landscape of death causes may have changed in metastatic NSCLC. We aim to compare patterns of death causes in metastatic NSCLC between the pre-immunotherapy and immunotherapy era to identify the consequent landscape transition of death causes.

**Methods:**

In this cohort study, 298,485 patients with metastatic NSCLC diagnosed between 2000 and 2018 were identified from the Surveillance, Epidemiology, and End Results Program. Unsupervised clustering with Bayesian inference method was performed for all patients’ death causes, which separated them into two death patterns: the pre-immunotherapy era group and the immunotherapy era group. Relative risk (RR) of each death cause between two groups was estimated using Poisson regression. Reduced death risk as survival time was calculated with locally weighted scatterplot smooth (Lowess) regression.

**Results:**

Two patterns of death causes were identified by unsupervised clustering for all patients. Thus, we separated them into two groups, the immunotherapy era (2015-2017, N=40,172) and the pre-immunotherapy era (2000-2011, N=166,321), in consideration of obscure availability to immunotherapy for patients diagnosed in 2012-2014, when the follow-up cutoff was set as three years. Although all-cause death risk had reduced (29.2%, 13.7% and 27.8% for death risks of lung cancer, non-cancer and other cancers), non-cancer deaths in the immunotherapy era (N=2,100, 5.2%; RR=1.155, 95%CI: 1.101-1.211, P<0.001) significantly increased than that in the pre-immunotherapy era (N=7,249, 5.0%), which included causes of chronic obstructive pulmonary disease, cerebrovascular disease, pneumonia and influenza, septicemia, infectious diseases, accidents and adverse effects, hypertension, and chronic liver disease and cirrhosis. However, cancer-caused deaths (excluding lung cancer) had no significant changes.

**Conclusions:**

The real-world landscape of death causes has changed in metastatic NSCLC when entering the immunotherapy era, and the increased non-cancer diseases may contribute to the changes that may be associated with commonly occurring irAEs.

## Introduction

Immunotherapy has gradually become one of the most important strategies to fight against malignancies, especially in patients with advanced or metastatic diseases, since the approval of the first immune checkpoint inhibitor (ICI), ipilimumab, by the US Food and Drug Administration in 2011. Current ICI treatments represented by monoclonal antibodies targeting immunosuppressive pathways including programmed death 1/programmed death ligand-1 and cytotoxic T-lymphocyte antigen-4 (CTLA-4), are unprecedentedly remodeling the therapeutic landscape of cancer by reactivating adaptive immunity (1, 2). In metastatic non-small cell lung cancer (NSCLC), anti-PD-1/PD-L1 treatments have made great achievements in improving patients’ survival outcomes, and have successfully advanced into frontline therapy (3, 4).

Despite great anti-cancer efficacy and generally-accepted tolerance, ICI treatments inevitably bring various degrees of inflammatory tissue damages due to excessive immune activation, referred to as immune-related adverse events (irAEs) (5). These irAEs are usually onset-delayed and unpredictable, demonstrating distinct characteristics from those of previous cancer therapies. According to the Common Terminology Criteria for Adverse Events (CTCAE) from the US National Cancer Institute, irAEs can be divided into five ranks (mild, moderate, severe, life-threatening and death). In some severe cases, a life-threatening event can develop, which may end up in death or irreversible pathological conditions. In a meta-analysis including 36 clinical trials, irAEs occurred approximately in 54%-76% of patients receiving ICI treatments (6). It can be inferred that the widespread use of ICI treatments is reshaping the landscape of death causes in metastatic cancer patients. Hence, it is highly necessary to promote rational use of ICI to avoid irAEs, rather than merely to pursue efficacy theoretically.

In the present study, we first did a real-world exploration of death causes in the immunotherapy era in patients with metastatic NSCLC, using the population-based cancer data from the Surveillance, Epidemiology, and End Results (SEER) database. We aimed to identify landscape transition of death causes when it shifted from the pre-immunotherapy time to the current immunotherapy era, and provide clinical references for rational medical management and more attentions on irAEs and non-cancer diseases among cancer survivors.

## Methods

### Patient source

The SEER Project initiated by National Cancer Institute has collected information of cancer patients that accounts for approximately 28% of the U.S. population. Metastatic NSCLC patients diagnosed between 2000 and 2018 were identified with definite records of follow-up. The exclusion criteria were as follows: 1) localized or regional disease at initial diagnosis (stage I, II, or III); 2) small cell type (histology codes: 8002/3, 8041/3, 8043/3, 8044/3, 8045/3, 8073/3 and 8803/3); 3) age at diagnosis less than 18 years old; and 4) unknown death causes, lost follow-up or died at diagnosis.

### Unsupervised clustering

Unsupervised clustering was performed for all deaths (2000-2018) to identify landscape changes of death causes as the diagnosis year. Each survival month with specific year was set as one sample dot for clustering. Features of death causes within each month were identified and assigned with the corresponding weighted proportions. The Bayesian inference method was used to quantify the features and weaken the side effects of the survival month itself, since we aimed to reveal the changes of death causes as diagnosis year. The central idea of the Bayesian method is the use of data to update the state of knowledge about a quantity of interest (7), which has been applied in most areas of medical statistics. Based on previous studies (8–10), the Bayesian inference procedures for this study were as follows:

Bayesian inference equation:


P(θ|data)=P(data|θ)P(θ)/P(data)∝P(data|θ)P(θ)

Firstly, for each specific feature, we took the Beta Distribution as the prior distribution:


$$\theta_{ij}∼Beta\left(\begin{array}{cc} N_{j}\Theta_{i}, & N_{j}-N_{j}\Theta_{i} \end{array}\right)$$

$$P\left(\theta_{ij}\right)=f_{Be}\left(\begin{array}{cc} \theta_{ij}; & N_{j}\Theta_{i}, \end{array}N_{j}-N_{j}\Theta_{i}\right)$$

where *i* and *j* corresponded to the diagnosis year and the survival month, respectively.

Define:


$$N_{j}=\bar{n_{ij}}$$

$$\Theta_{i}=\sum_{j}m_{ij}/\sum_{j}n_{ij}$$

where *n*_*ij*_ represented the total number of deaths and *m*_*ij*_ represented the number of cases that died of the corresponding cause in each sample dot.

Then, the posterior distribution was:


$$\theta_{ij}|data∼Beta\left(N_{j}\Theta_{i}+m_{ij},N_{j}-N_{j}\Theta_{i}+n_{ij}-m_{ij}\right)$$

$$P\left(\theta_{ij}|data\right)=f_{Be}\left(\begin{array}{cc} \theta_{ij}; & N_{j}\Theta_{i}+m_{ij}, \end{array}N_{j}-N_{j}\Theta_{i}+n_{ij}-m_{ij}\right)$$

Finally, the expectation for each specific feature of each survival month was:


$$\overset{\wedge }{\theta_{ij}}=E\left(\theta_{ij}|data\right)=\frac{N_{j}\Theta_{i}+m_{ij}}{N_{j}+n_{ij}}$$

among which, that is to say, *θ* represented the possibility of dying from certain cause for individual patient with certain survival (months) and a certain diagnosis year.

K-means clustering algorithm was used for analyzing the pattern changes of death causes from 2000 to 2018. Uniform Manifold Approximation and Projection (UMAP) method was conducted for dimensionality reduction and visualization. Before clustering, the samples that included less than 20 cases were excluded during the pre-processing.

### Statistical analysis

Patient data was extracted from SEER 18 Registries Database using SEER*Stat version 8.3.9. The OS curve was plotted using Kaplan-Meier method. To find the distribution pattern changes of death causes, Poisson regression analysis was performed to estimate the relative risk (RR) with 95% confidence interval (95%CI) of each death cause among all deaths of the immunotherapy group compared to those of the pre-immunotherapy group, with adjustments by potential confounding baseline characteristics: sex, age and race.

The risks of lung cancer death, non-cancer death and non-lung cancer death (all cancer deaths but excluding those caused by lung malignancies) at each survival month were expressed as the ratio of the number of deaths during the period to the number of patients at risk (who were alive at the beginning of the month). Furthermore, locally weighted scatterplot smooth (Lowess) regression analysis was used to visualize the risks of each death cause as the survival month. The total risk change from the pre-immunotherapy time to the immunotherapy era was estimated using areas under the Lowess curve (AUCs) of the immunotherapy group minus the pre-immunotherapy group. We compared the risks between the two groups by Wilcoxon matched-pairs signed rank test.

Excess deaths of each non-cancer death cause (per 100,000 deaths) related to the immunotherapy era were calculated by taking the number of deaths in the immunotherapy group minus the number of those in the pre-immunotherapy group among subpopulations of different genders, ages and races. P value<0.05 was considered statistically significant.

## Results

### Clustering analysis identifies two patterns of death causes

**Figure 1:**
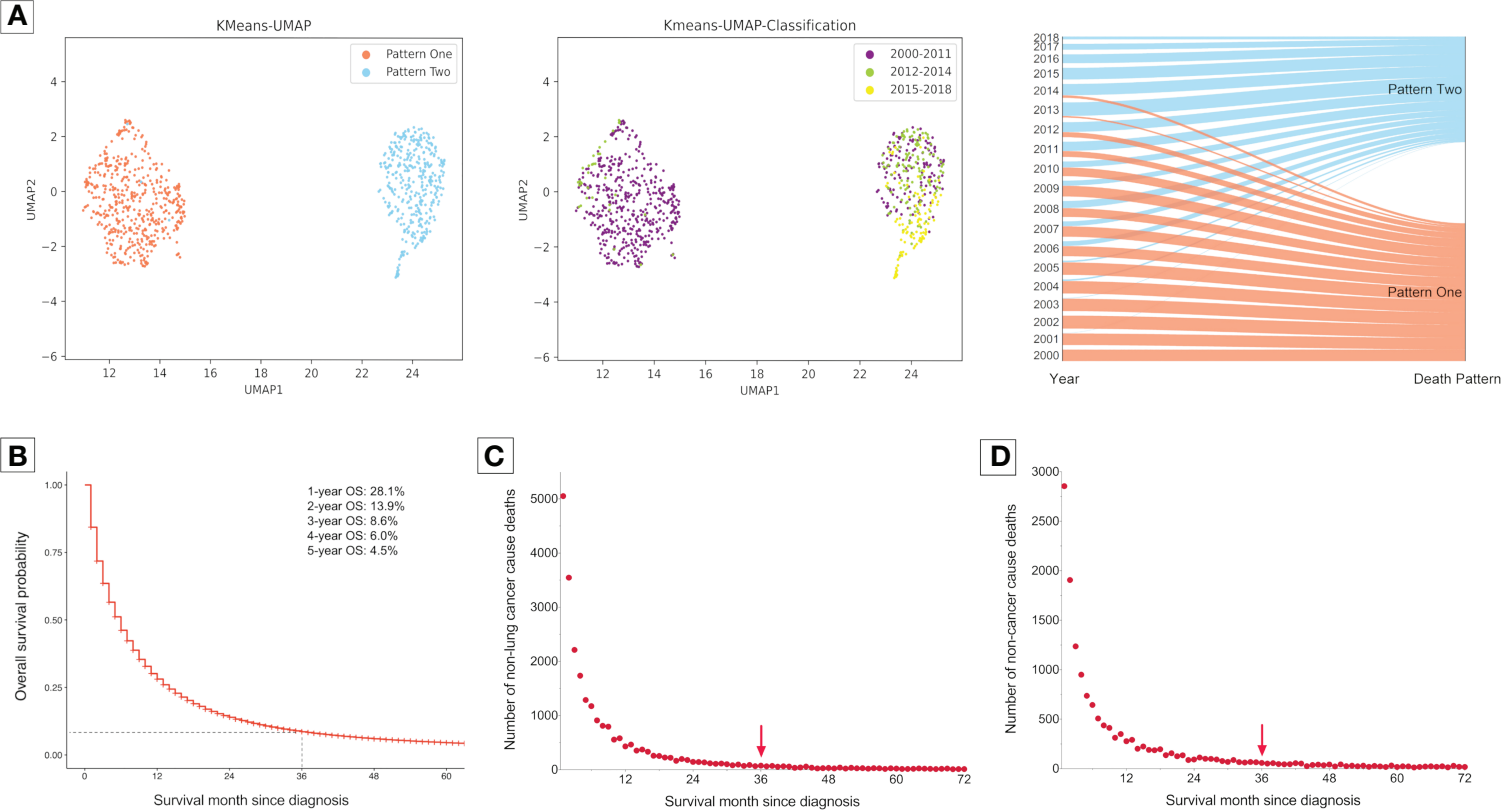
Clustering analysis for grouping of death patterns and identifying the optimal cutoff of follow-up time in the subsequent analyses in 298,485 patients with metastatic non-small cell lung cancer. Patients were diagnosed between 2000 and 2018 form the Surveillance, Epidemiology, and End Results Program. **(A)**, Clustering presentations. **(B)**, Overall survival probability (OS) curve for 298,485 patients with metastatic non-small cell lung cancer diagnosed between 2000 and 2018. Most of them died within three years after diagnosis. **(C)**, Number of deaths caused by cancers but excluding lung cancer with survival month. **(D)**, Number of deaths caused by non-cancer diseases with survival month. There were very few deaths caused by non-lung cancers and non-cancer diseases three years after diagnosis.

The clustering analysis for a total of 298,485 metastatic NSCLC patients who had died until the last follow-up (November 2021) identified two obviously isolated distribution patterns of death causes according to the diagnosis year (**Figure 1A**). Most of the patients who were diagnosed in 2000-2011 gathered together in Pattern One, while those diagnosed in 2015-2018 were in Pattern Two. For patients diagnosed in 2012-2014, the sample dots existed in both death patterns.

**Figure 2:**
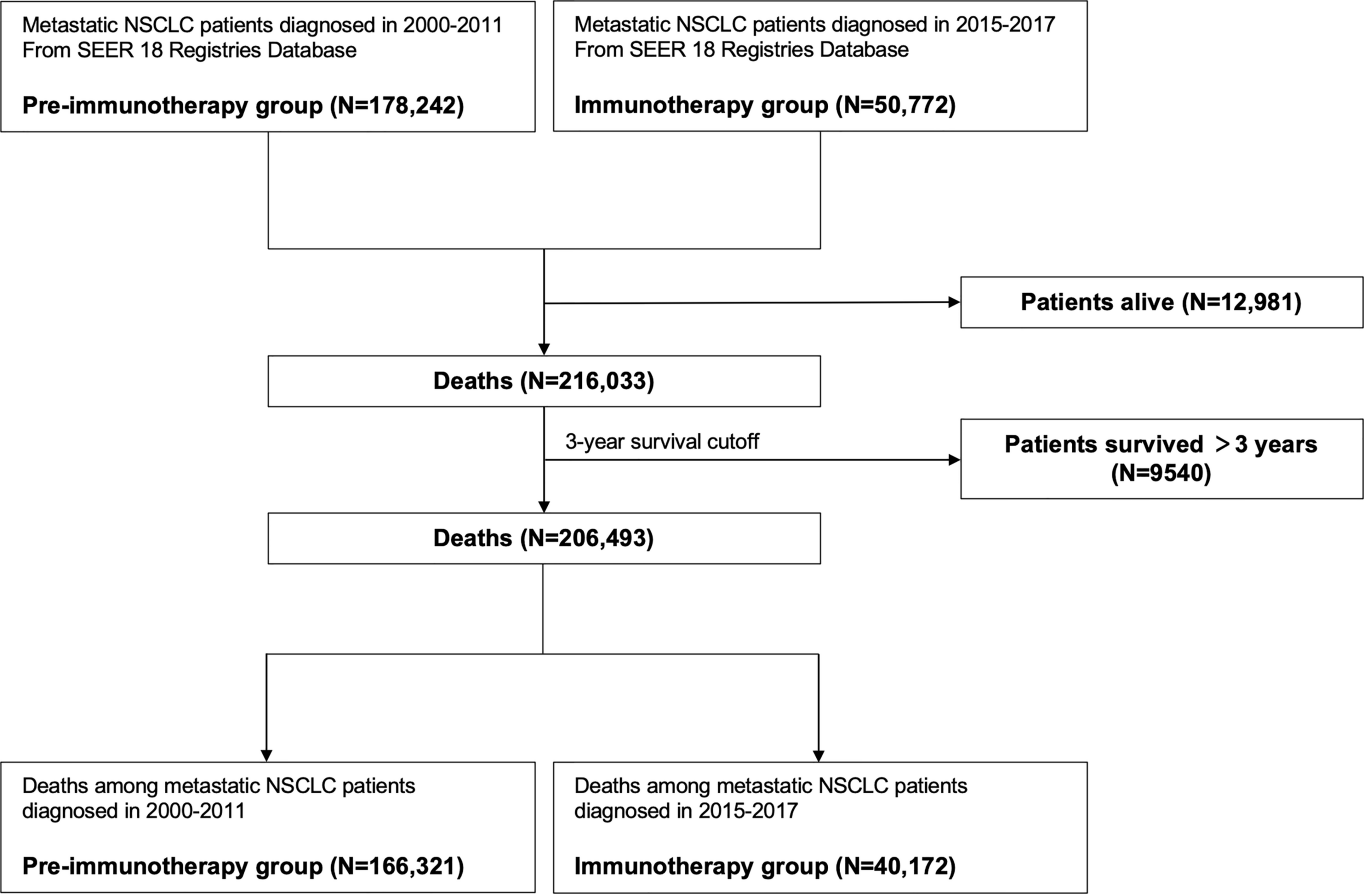
Flowchart of identifying patients with metastatic non-small cell lung cancer for grouping of the pre-immunotherapy era and the immunotherapy era.

Next, we aimed to investigate the pattern changes of death causes in the immunotherapy era among metastatic NSCLC patients. To avoid bias related to longer follow-up time for those who were diagnosed earlier, we only enrolled patients that died within three years after diagnosis, since most of them had met death during the period (3-year overall survival probability: 8.6%; **Figure 1B**). Moreover, there were very few deaths of non-cancer (**Figure 1C**) or non-lung cancer (died of cancer causes but excluding lung cancer; **Figure 1D**) causes three years after NSCLC diagnosis. Furthermore, considering the death pattern ambiguity of cases diagnosed in 2012-2014, we excluded them in the subsequent analyses, which, meanwhile, ensured that the pre-immunotherapy group had no availability to immunotherapy (approved for NSCLC in 2015). Thus, we divided all deaths into two groups, the immunotherapy era (2015-2017) and the pre-immunotherapy era (2000-2011) (**Figure 2**).

### Proportion change of death causes attributable to the immunotherapy era

**Figure 3:**
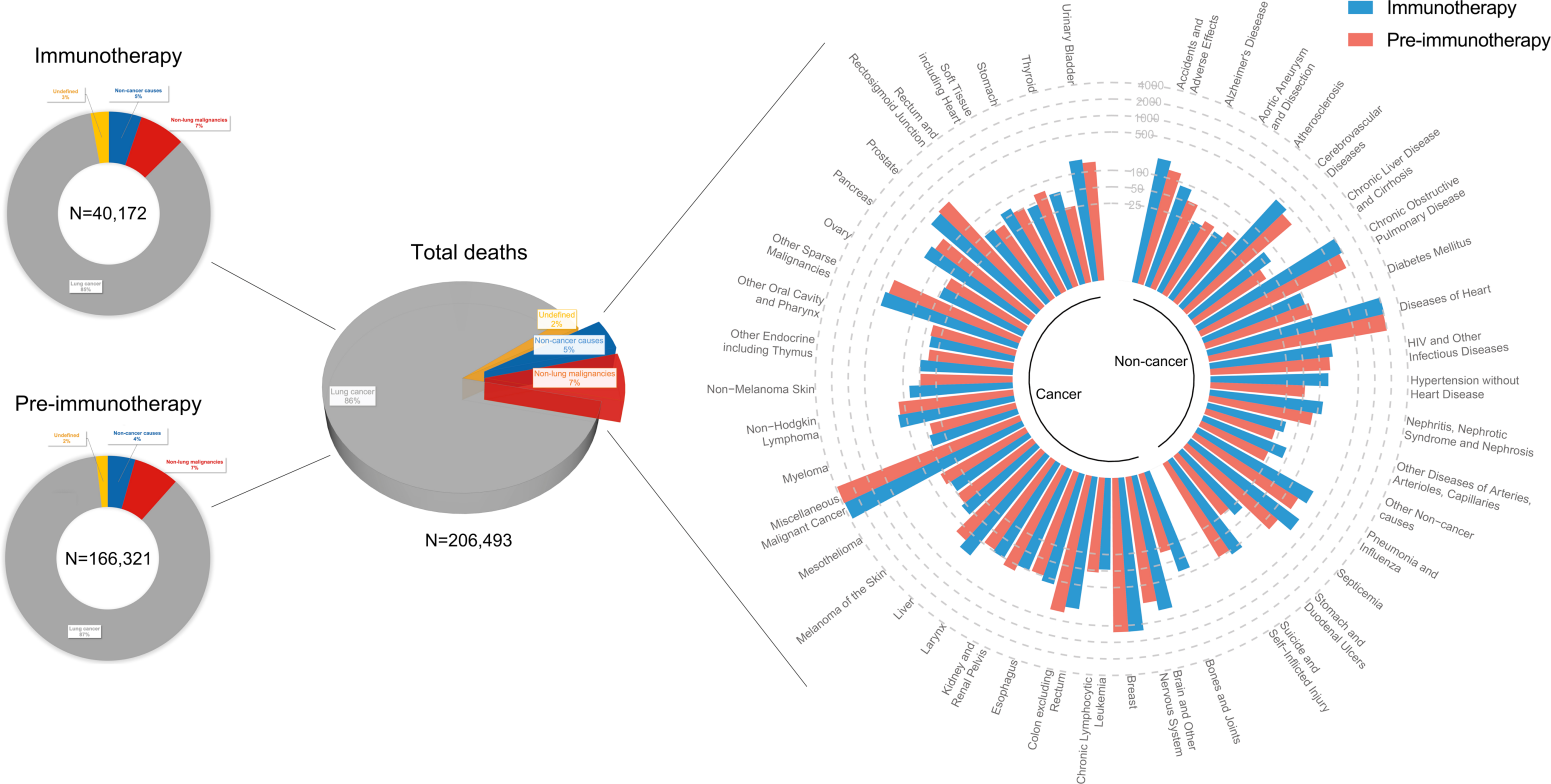
Landscapes of death causes in the pre-immunotherapy era (2000-2011, N=166,321) and in the immunotherapy era (2015-2017, N=40,172) for patients with metastatic non-small cell lung cancer. Number of deaths at each death cause was presented except lung cancer. Approximately 2% of them (N=4,215) had no definitive records of death causes.

**Figure 4:**
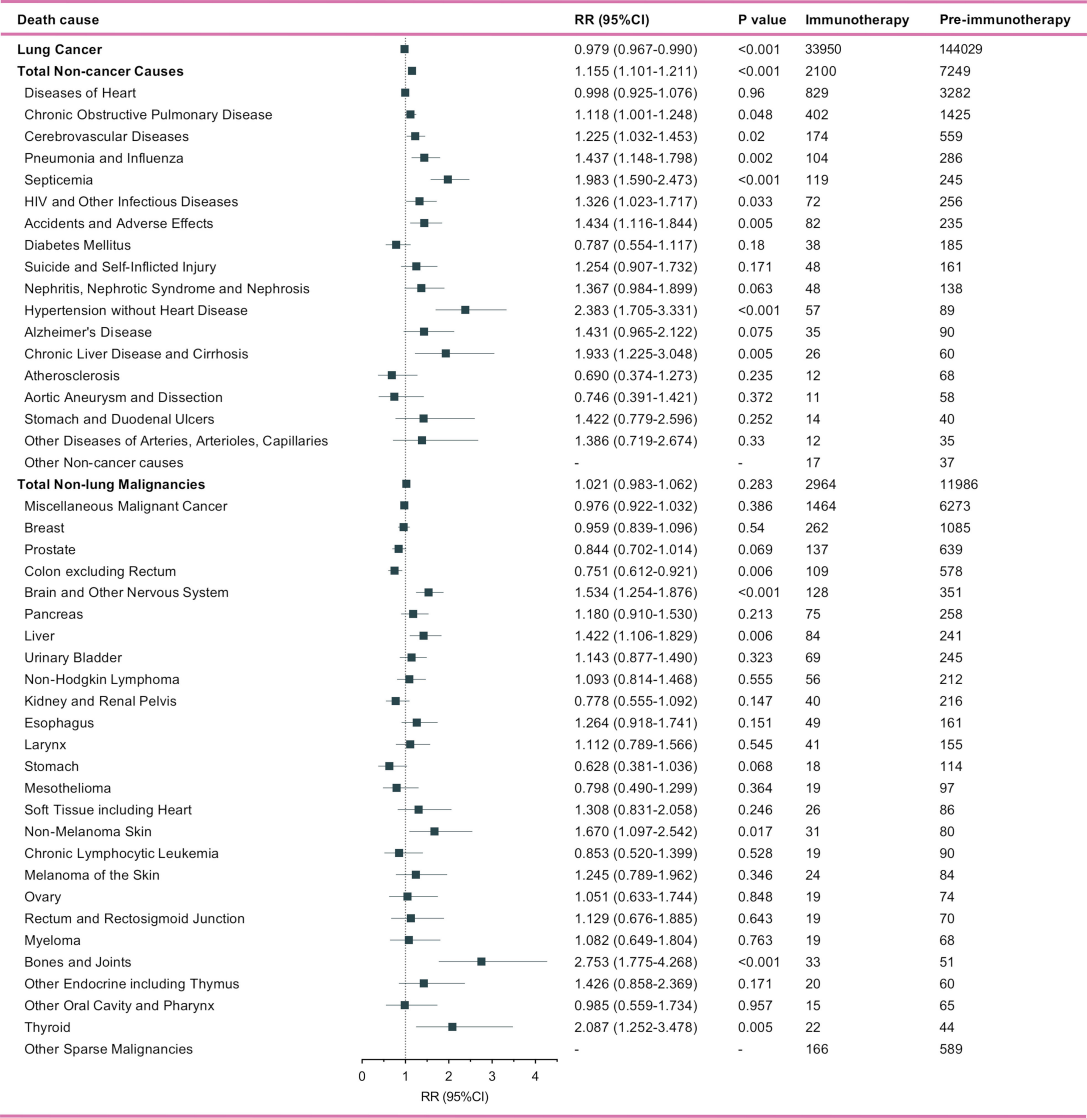
Number and relative risk (RR) with 95% confidence interval (95%CI) of deaths at each death cause between pre-immunotherapy era and the immunotherapy era in patients with metastatic non-small cell lung cancer. Patients in the pre-immunotherapy era group were as reference.

Of metastatic NSCLC patients, a total of 166,321 and 40,172 deaths were identified into the pre-immunotherapy group (2000-2011) and the immunotherapy group (2015-2017), respectively (**Figure 3**). During the 3-year cutoff follow-up, 84.5% (N=33,950) of deaths in the immunotherapy died of lung cancer with a significant RR of 0.979 (95%CI: 0.967-0.990, P<0.001), when compared with 86.6% (N=144,029) of those in the pre-immunotherapy group (**Figure 4**). However, the proportion of non-cancer deaths in the immunotherapy group (N=2,100, 5.2%; RR=1.155, 95%CI: 1.101-1.211, P<0.001) was significantly higher than that in the pre-immunotherapy group (N=7,249, 5.0%), which specifically included chronic obstructive pulmonary disease (COPD; RR=1.118, 95%CI: 1.001-1.248, P=0.048), cerebrovascular disease (RR=1.225, 95%CI: 1.032-1.453, P=0.020), pneumonia and influenza (RR=1.437, 95%CI; 1.148-1.798, P=0.002), septicemia (RR=1.983, 95%CI: 1.590-2.473, P<0.001), other infectious diseases including HIV (RR=1.326, 95%CI: 1.023-1.717, P=0.033), accidents and adverse effects (RR=1.434, 95%CI: 1.116-1.844, P=0.005), hypertension without heart disease (RR=2.383, 95%CI: 1.705-3.331, P<0.001), and chronic liver disease and cirrhosis (RR=1.933, 95%CI: 1.225-3.048, P=0.005).

**Figure 5:**
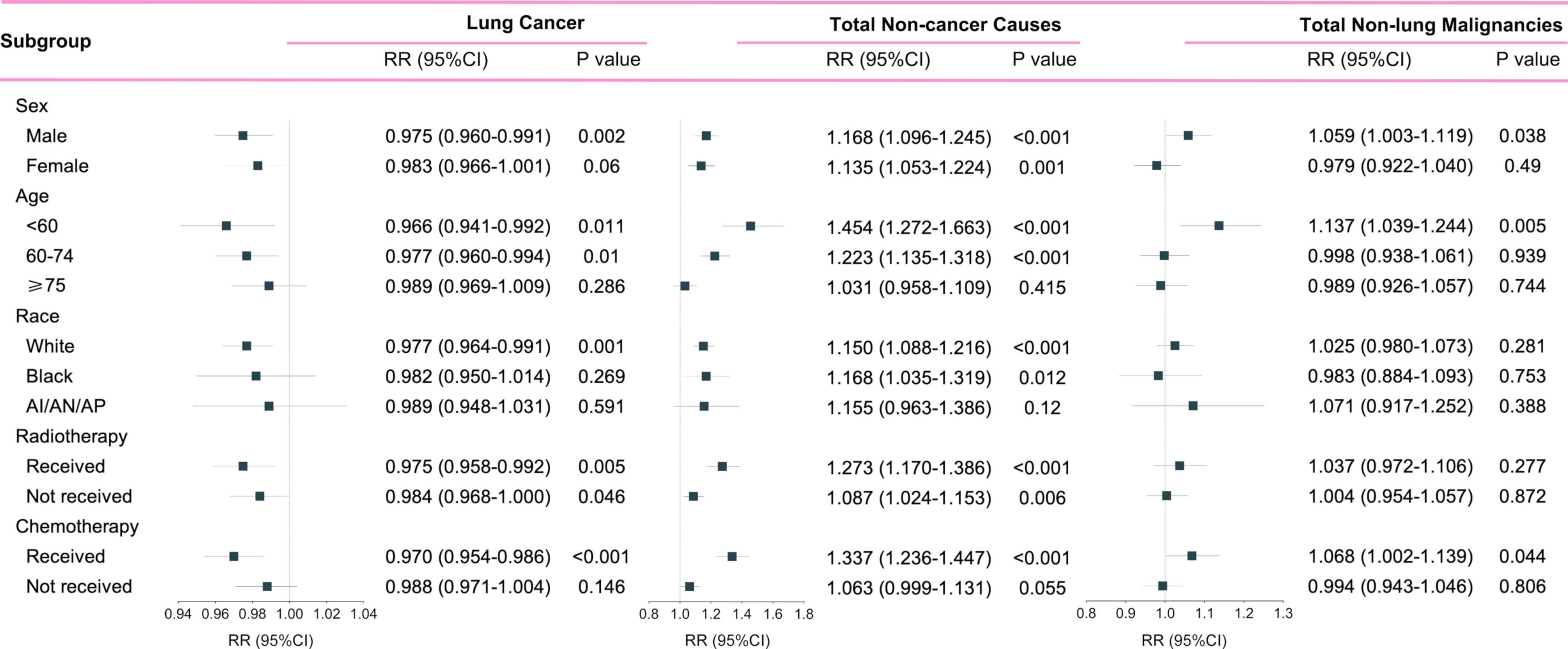
Subgroup analyses of relative risk (RR) with 95% confidence interval (95%CI) of deaths at each death cause between pre-immunotherapy era and the immunotherapy era in patients with metastatic non-small cell lung cancer. Patients in the pre-immunotherapy era group were as reference. AI/AN/AP, American Indian/Alaska Native/Asian and Pacific Islander.

Patients who died of other primary malignancies showed insignificant changes in their proportion among all deaths when transferring from the pre-immunotherapy time (N=11,986, 7.2%) to the recent immunotherapy era (N=2,964, 7.4%; RR=1.021, 95%CI: 0.983-1.062, P=0.283). In detail, however, malignancies of nervous system (RR=1.534, 95%CI: 1.254-1.876, P<0.001), liver (RR=1.422, 95%CI: 1.106-1.829, P=0.006), skin excluding melanoma (RR=1.670, 95%CI: 1.097-2.542, P=0.017), bones and joints (RR=2.753, 95%CI: 1.775-4.268, P<0.001), and thyroid (RR=2.087, 95%CI: 1.252-3.478, P=0.005) were found to have significantly higher proportions among all deaths, while the colon cancer demonstrated an opposite change (RR=0.751, 95%CI: 0.612-0.921, P=0.006). Additionally, the heterogeneity of proportion changes among different subgroups was also analyzed in **Figure 5**.

### Risk of death causes as survival time

**Figure 6:**
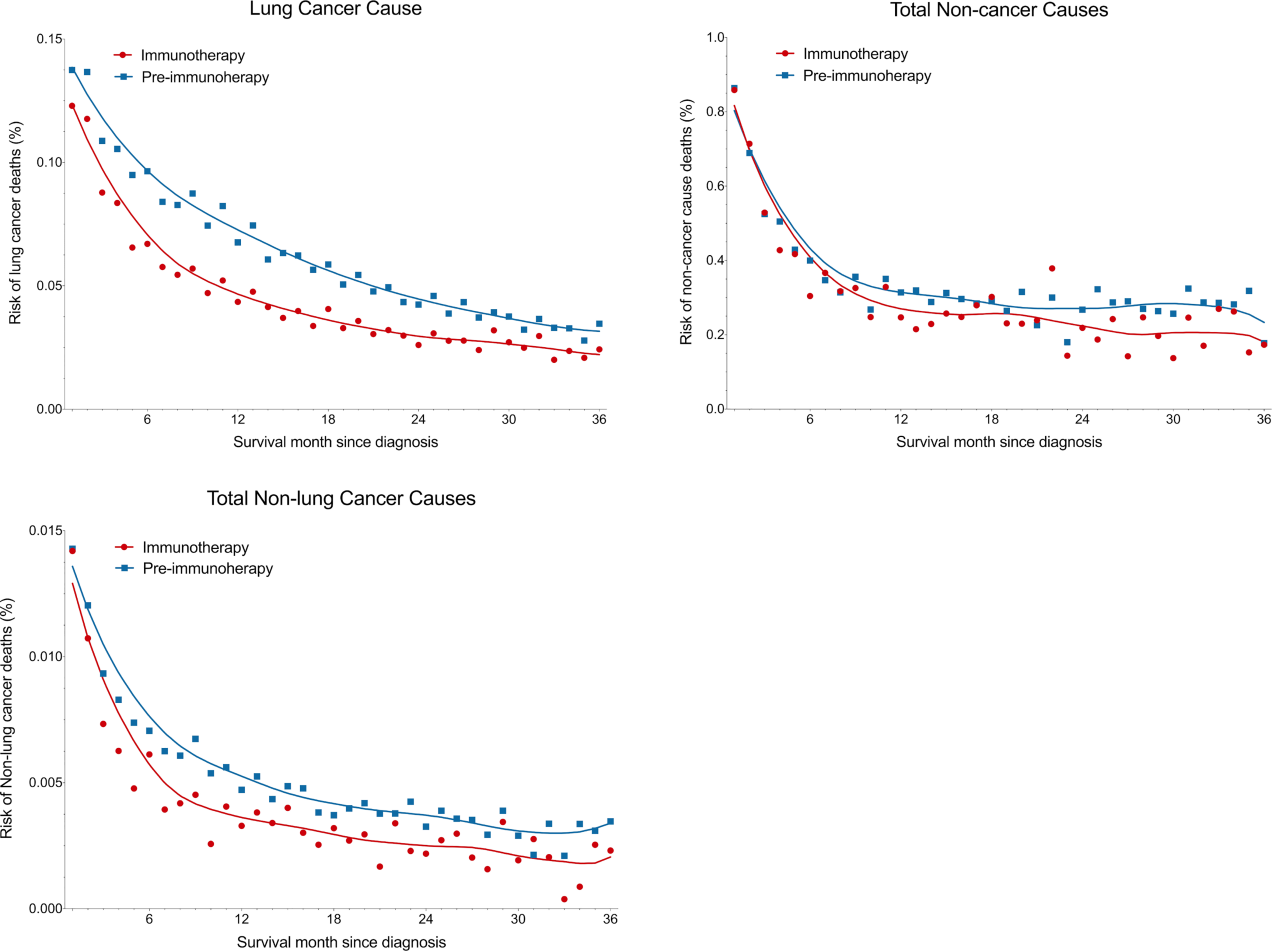
Reduced death risk at lung cancer, non-cancer and non-lung cancer causes in the immunotherapy era than in the pre-immunotherapy era in patients with metastatic non-small cell lung cancer. Overall three-year death risks for lung cancer, non-cancer and non-lung cancer causes were reduced by 29.2%, 13.7% and 27.8%, respectively.

We observed that all risks of lung cancer cause, non-lung cancer causes, and non-cancer causes, decreased as the survival month increased (**Figure 6**). Metastatic NSCLC patients did have significantly lower death risks at all three kinds of causes in the immunotherapy era than before (all with P<0.001 by Wilcoxon matched-pairs signed rank test). Compared with the pre-immunotherapy time, the risks of lung cancer cause, non-cancer causes and non-lung cancer causes decreased by 29.2%, 13.7% and 27.8%, respectively (calculated by differences in AUCs of the fitted Lowess curves). As the survival month increased, the death risks started to decline quickly in the first year, especially for non-cancer causes, which demonstrated a nearly flat trend in the later two survival years. As expected, the risks of death caused by certain chronic diseases also significantly decreased based on more detailed analyses, such as diabetes mellitus (P=0.017), COPD (P=0.027) and diseases of heart (P<0.001) (**Figure S1**).

### Change of deaths at each non-cancer cause by subgroup analyses

**Figure 7:**
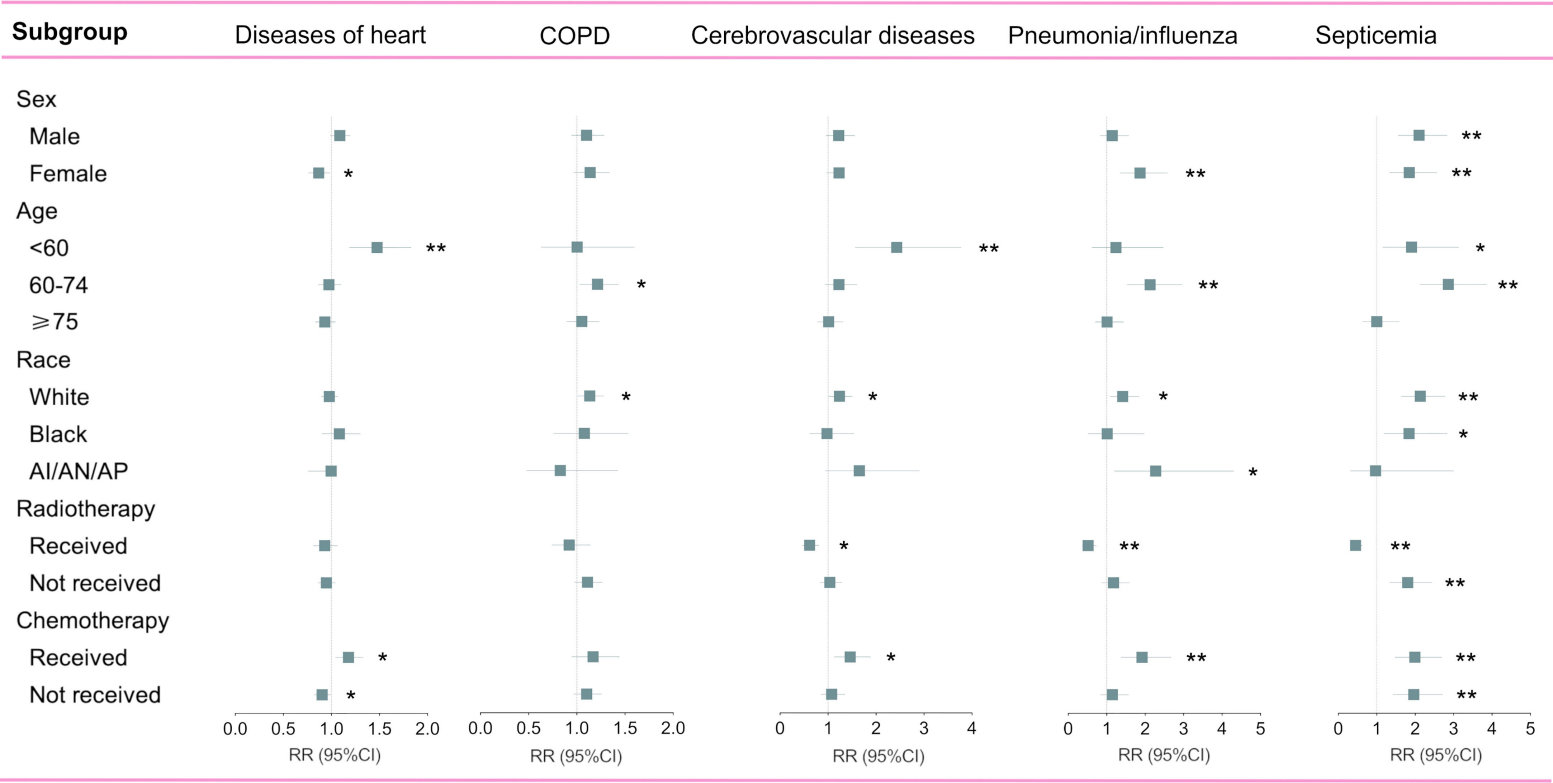
Subgroup analyses of relative risk (RR) with 95% confidence interval (95%CI) of deaths at each of top five non-cancer causes between pre-immunotherapy era and the immunotherapy era in patients with metastatic non-small cell lung cancer. Patients in the pre-immunotherapy era group were as reference. AI/AN/AP, American Indian/Alaska Native/Asian and Pacific Islander. *P value <0.05; **P value <0.01.

**Figure 8:**
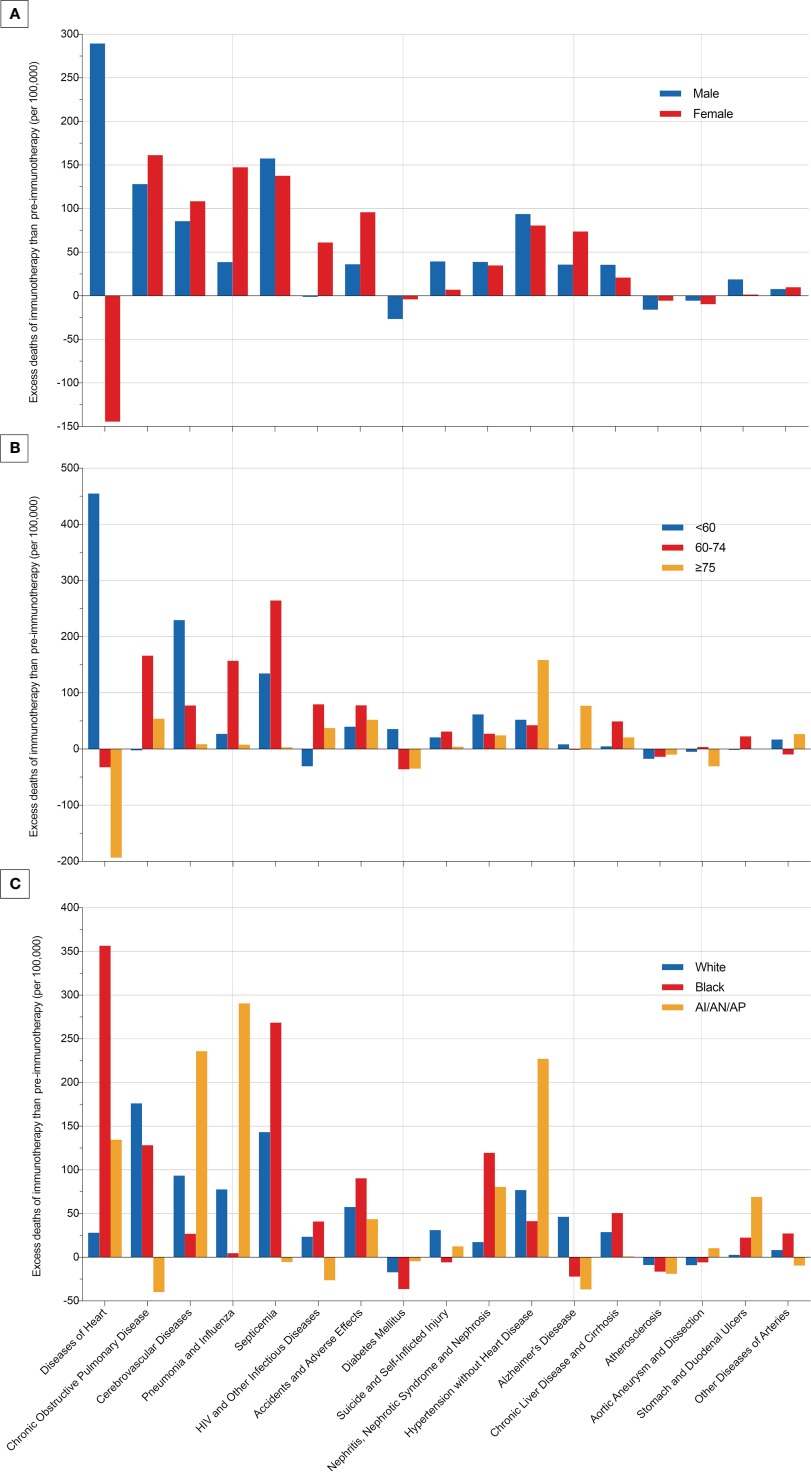
Excess deaths of each most common non-cancer cause per 100,000 patients with metastatic non-small cell lung cancer in the immunotherapy era than in the pre-immunotherapy era.

**Figure 9:**
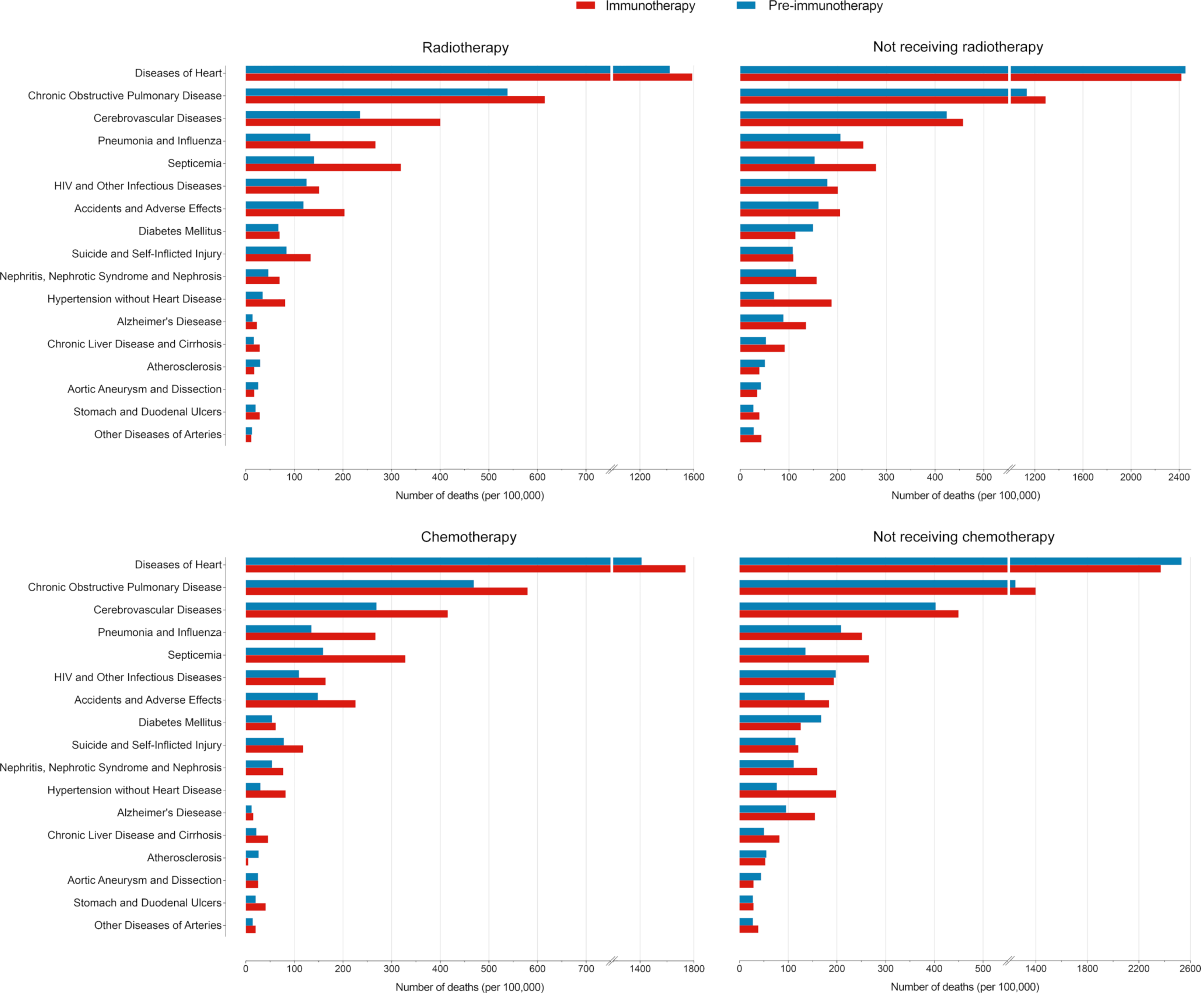
Comparisons of number of deaths per 100,000 patients with metastatic non-small cell lung cancer between the pre-immunotherapy and the immunotherapy eras grouped by treatments.

Since non-cancer deaths varied remarkably from the pre-immunotherapy to the immunotherapy era, we then performed subgroup analyses to reveal their change heterogeneity in different genders, ages and races. For example, even there was no significant change for the overall proportion of deaths caused by heart diseases among all deaths, subgroups of different genders demonstrated exactly reverse distribution changes, and females died of heart diseases significantly decreased in the immunotherapy era than before (RR=0.868, 95%CI: 0.764-0.985, P=0.028) while those died of pneumonia/influenza significantly increased (RR=1.869, 95%CI: 1.353-2.582, P<0.001; **Figure 7** and **Table S1**). Male deaths caused by heart diseases increased by 289 cases per 100,000 in the immunotherapy time than before, while female deaths decreased by 144 deaths per 100,000 (**Figure 8A**). Besides, we also observed that patients aged<60 years showed a significant increase in heart (RR=1.476, 95%CI: 1.190-1.829, P<0.001) and cerebrovascular (RR=2.43, 95%CI: 1.567-3.769, P<0.001) diseases-caused deaths in the immunotherapy era, compared with those aged ≥60 years who seemed to have an opposite change in these deaths (**Figure 8B**). Furthermore, though without significant changes in most subgroup analyses for non-white populations, which accounted for relatively small proportions, the black (357 cases per 100,000) had the largest excess number of heart diseases-caused deaths from the pre-immunotherapy to the immunotherapy era, followed by American Indian Alaska Native/Asian and Pacific Islander (AI/AN/AP, 134 cases per 100,000), while white people merely had a small increase (28 cases per 100,000; **Figure 8C**). As for treatments, we observed that proportions of deaths caused by cerebrovascular diseases (RR=0.615, 95%CI: 0.466-0.812, P=0.001), pneumonia/influenza (RR=0.517, 95%CI: 0.365-0.733, P<0.001) and septicemia (RR=0.450, 95%CI: 0.325-0.622, P<0.001) had significantly reduced among patients receiving radiotherapy in the immunotherapy era. However, patients receiving chemotherapy seemed to have an opposite change trend, and proportions of deaths caused by heart diseases (RR=1.178, 95%CI: 1.044-1.330, P=0.008), cerebrovascular diseases (RR=1.461, 95%CI: 1.132-1.885, P=0.004), pneumonia/influenza (RR=1.919, 95%CI: 1.379-2.671, P<0.001) and septicemia (RR=1.991, 95%CI: 1.475-2.688, P<0.001) significantly increased, probably suggesting the additional toxicity of chemotherapy combined immunotherapy (**Figures 7**, **9**; **Table S1**).

## Discussion

The immunotherapy representative by ICIs has revolutionized the treatment pattern for cancer with encouraging survival benefits, but may be accompanied by adverse events, especially lethal irAEs (11). Although several guidelines have been proposed for the management of these events (11–14), how the novel alternative treatment affects the pattern of death causes remains unclear. Using the largest cancer database of the SEER, this study firstly identified the landscape transition of death causes among metastatic NSCLC patients from the pre-immunotherapy time to the immunotherapy era. For all 298,485 deaths with metastatic NSCLC diagnosed between 2000 and 2018, we observed two obvious clustering patterns of death distributions, between which the cutoff period approached the time when ICIs treatment was approved. Although the total death risk has significantly decreased, the proportions of death causes demonstrated discrete changes in the immunotherapy era. Specifically, non-cancer causes deaths were found to have a significantly higher proportion than before. Therefore, we hypothesize that immunotherapy may partly attribute to these pattern changes of death causes among metastatic NSCLC patients by facilitating adverse events directly or indirectly.

With rapid advances in the treatment of lung cancer, NSCLC patients’ survival has been dramatically improved with the decreased death rates. As expected, our results consistently demonstrated a significantly lower death risk for the lung cancer cause, total non-cancer causes and total non-lung malignancies in the immunotherapy era when compared with those in the pre-immunotherapy time. However, the reduced risk of non-cancer causes was less than half that of cancer causes. Besides, we also observed a significantly higher proportion of non-cancer deaths with an increased relative risk by 15.5%. Therefore, as the overall mortality gradually declines with the survival outcome improved, the management of other chronic diseases may require more concerns when entering the immunotherapy era, such as COPD, cerebrovascular disease, hypertension, chronic liver disease and cirrhosis, and so on.

In detail, the subgroup analyses of death proportions revealed the potential heterogeneity of changes among metastatic NSCLC patients. Firstly, male patients showed a significantly decreased proportion of the death cause of lung cancer but a significantly increased proportion of the death cause of other malignancies, while females had no such significant changes. The decreased lung cancer deaths in males may indicate the sex disparity of the immunotherapy’s efficacy on metastatic NSCLC, which was consistent with the previous study that male patients with advanced or metastatic cancer had better efficacy than females when treated with ICIs (15). As for age, younger patients were inclined to have more reductions in the proportion of lung cancer-caused deaths, but they increased more in the relative death risk of other causes (non-cancer and non-lung malignancies). Young patients usually have better physical states and lower risk of non-cancer diseases than the older, and thus the risk increase of non-cancer death causes among them may be more obvious. It is also necessary to pay more attentions to irAEs in young patients receiving immunotherapy. The strategy of ICIs combined with chemotherapy (16, 17) or radiotherapy (18) could bring more therapeutic effects, but it would also cause more toxicities, in accordance with our results that patients receiving radiotherapy or chemotherapy demonstrated more decreases in the proportion of lung cancer deaths and more increases in the proportion of non-cancer deaths than those who did not receive in the immunotherapy era.

Though the overall proportion of cardiac deaths showed no significant changes between the immunotherapy and the pre-immunotherapy eras, we observed different change trends in the number among subgroups by sex and age. Deaths caused by heart diseases severely rose in the immunotherapy era among male and young (<60 years old) patients. We speculate that the novel immunotherapy represented by ICIs may partly attribute to these disparities. Firstly, in the whole population, the death risk of heart diseases was supposed to gradually decrease for both sexes, though females were thought to have a lower risk (19). Secondly, immune-related cardiotoxic events occurred commonly in young patients, thus attributable to the surprising increase of cardiac deaths in the immunotherapy era (20–22). Previous evidence has revealed the associations between immunotherapy and higher cardiotoxicity-related mortality (23), and patients who died of cardiac events also showed worse overall survival recently than those in the pre-immunotherapy time (24).

Besides, we also observed consistently higher proportions of infectious causes’ deaths in the immunotherapy, including pneumonia, influenza, septicemia, HIV, and other infectious diseases. Although Tong et al. suggested that PD-1/PD-L1 inhibitors significantly increased the risk of all-grade and high-grade pneumonia in NSCLC patients (25), the mechanism of how ICIs affect cancer patients’ immune status against infections remains unclear.

Several limitations are required to be declared for this study. Firstly, patients’ clinical data from the SEER database did not include the details of treatments. Secondly, this is a retrospective study and cannot accurately analyze all factors that are attributed to the landscape changes of death causes among metastatic NSCLC patients. Thirdly, the classification criteria of death cause in the SEER database are based on International Classification of Diseases (ICD-10) recodes, and thus cannot investigate more delicate classification information of death causes, such as immune-related myocarditis.

## Conclusions

Our study firstly identified the real-world landscape transition of death causes among metastatic NSCLC patients from the pre-immunotherapy time to the immunotherapy era. Despite the decreased overall death risk, the proportion of non-cancer causes significantly increased among all deaths, which can be partly attributed to the increased non-cancer diseases that may be associated with commonly occurring irAEs, highlighting the importance of rational ICI uses, and of focusing more on non-cancer diseases in patients receiving immunotherapy.

## Data availability statement

The original contributions presented in the study are included in the article/**Supplementary Material**. Further inquiries can be directed to the corresponding authors.

## Ethics statement

The studies involving human participants were reviewed and approved by Ethics Committee of West China Hospital. The patients/participants provided their written informed consent to participate in this study.

## Author contributions

YW and ZY had full access to all of the data in the study and take responsibility for the integrity of the data and the accuracy of the data analysis. Concept and design: YW, ZY, KK, AZ. Acquisition, analysis, or interpretation of data: YW, ZY, AZ, CH. Drafting of the manuscript: YW, ZY. Critical revision of the manuscript for important intellectual content: All authors. Statistical analysis: YW, ZY, AZ, KK. Obtained funding: ZY, AZ. Administrative, technical, or material support: YW, ZY, CH. Supervision: KK, AZ. All authors contributed to the article and approved the submitted version.

## Funding

This work was supported by Post-Doctor Research Project, West China Hospital, Sichuan University (No. 2020HXBH101), “from zero to one” Innovation Research Project of Sichuan University (No. 2022SCUH0025), Chengdu Science and Technology Program (No. 2022-YF05-01443-SN), China Postdoctoral Science Foundation (No. 2021M692310), and National Natural Science Foundation of China (No. 82204490, No. 82203022).

## Conflict of interest

The authors declare that the research was conducted in the absence of any commercial or financial relationships that could be construed as a potential conflict of interest.

## Publisher’s note

All claims expressed in this article are solely those of the authors and do not necessarily represent those of their affiliated organizations, or those of the publisher, the editors and the reviewers. Any product that may be evaluated in this article, or claim that may be made by its manufacturer, is not guaranteed or endorsed by the publisher.

## Supplementary material

Supplementary Figure 1 Reduced death risk at each most common non-cancer cause in the immunotherapy era when compared with those in the pre-immunotherapy era for patients with metastatic non-small cell lung cancer.
