# Erratum: Real-world landscape transition of death causes in the immunotherapy era for metastatic non-small cell lung cancer

**DOI:** 10.3389/fimmu.2022.1114331

**Published:** 2022-12-12

**Authors:** Frontiers Production Office

**Affiliations:** Frontiers Media SA, Lausanne, Switzerland

**Keywords:** death cause analysis, immunotherapy, non-small cell lung cancer, metastatic disease, real world data

Due to a production error, there was a mistake on the first line of the Methods section where the number of patients with metastatic NSCLC was incorrectly captured as “298,48patients” when it should have been “298,485 patients”.

A correction has been made to the Abstract section, subsection Methods:

“In this cohort study, 298,485 patients with metastatic NSCLC diagnosed between 2000 and 2018 were identified from the Surveillance, Epidemiology, and End Results Program. Unsupervised clustering with Bayesian inference method was performed for all patients’ death causes, which separated them into two death patterns: the pre-immunotherapy era group and the immunotherapy era group. Relative risk (RR) of each death cause between two groups was estimated using Poisson regression. Reduced death risk as survival time was calculated with locally weighted scatterplot smooth (Lowess) regression.”

The publisher apologizes for this mistake.

The original version of this article has been updated.

