# Supplementary figures and images for: Real-world landscape transition of death causes in the immunotherapy era for metastatic non-small cell lung cancer
Source: Front Immunol. 2022 Nov 11;13:1058819. doi: 10.3389/fimmu.2022.1058819 (PMC9691859; doi:10.3389/fimmu.2022.1058819)

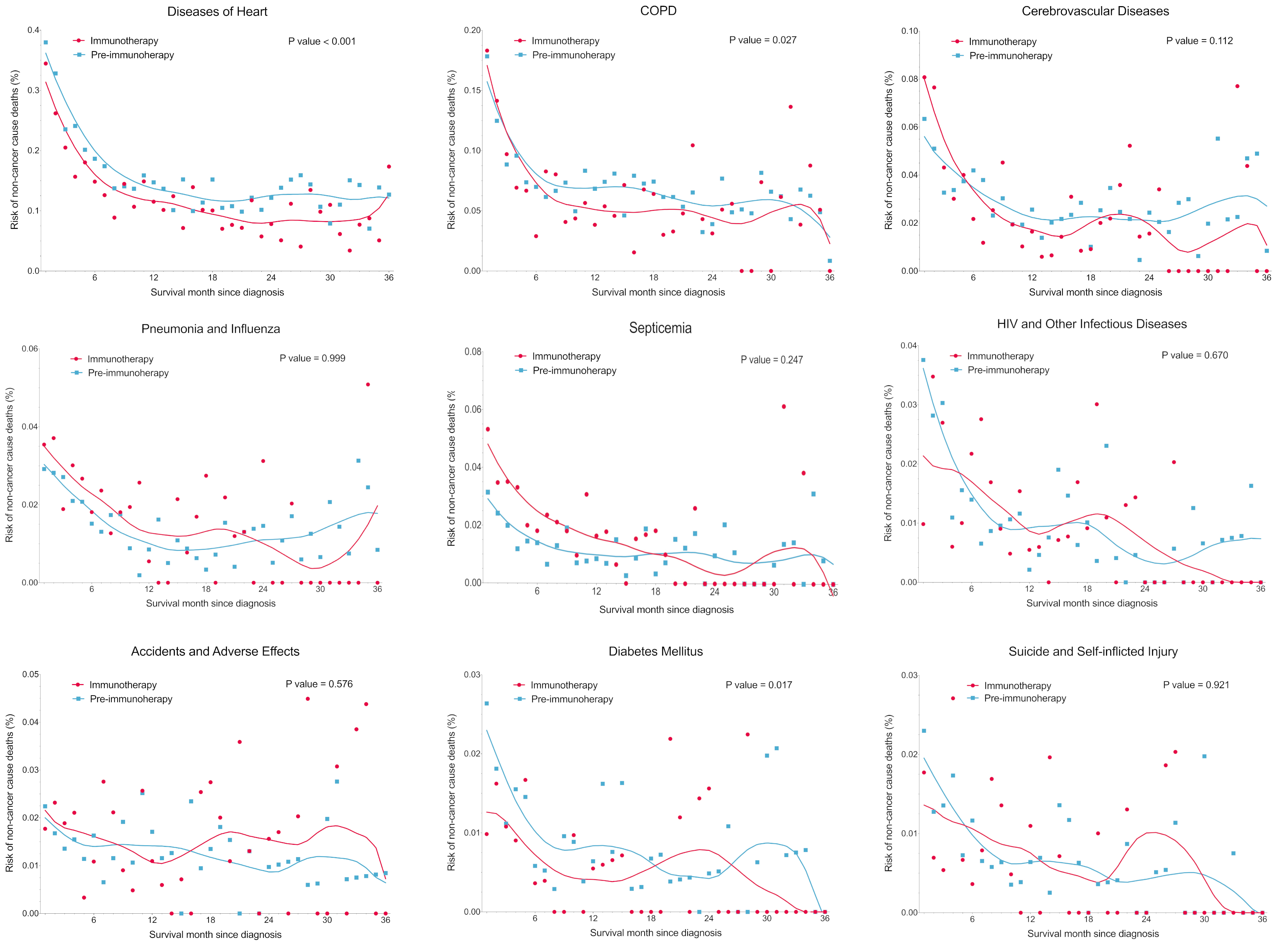

Supplement: Supplementary Figure 1 — Reduced death risk at each most common non-cancer cause in the immunotherapy era when compared with those in the pre-immunotherapy era for patients with metastatic non-small cell lung cancer. [file Image_1.jpeg]
